# Supplementary material for: Metabolic Reprogramming and Its Relationship to Survival in Hepatocellular Carcinoma
Source: Cells. 2022 Mar 22;11(7):1066. doi: 10.3390/cells11071066 (PMC8997969; doi:10.3390/cells11071066)
Supplement: Supplementary file 1 [file cells-11-01066-s001.zip › cells-1613950-supplementary.pdf]

Supporting Information

Metabolic Reprogramming and Its Relationship to Survival in Hepatocellular Carcinoma

Qingqing Wang [1,2], Yexiong Tan [3], Tianyi Jiang [3], Xiaolin Wang [1], Qi Li [1], Yanli Li [1], Liwei Dong [3,*], Xinyu Liu [1,*] and Guowang Xu [1]

[1] CAS Key Laboratory of Separation Science for Analytical Chemistry, Dalian Institute of Chemical Physics, Chinese Academy of Sciences, Dalian 116023, China; wangqq15@dicp.ac.cn (Q.W.); wangxiaolin@dicp.ac.cn (X.W.); liqi1808@dicp.ac.cn (Q.L.); liyanli@dicp.ac.cn (Y.L.); liuxy2012@dicp.ac.cn (X.L.); xugw@dicp.ac.cn (G.X.)

[2] University of Chinese Academy of Sciences, Beijing 100049, China

[3] International Cooperation Laboratory on Signal Transduction, Eastern Hepatobiliary Surgery Institute, The Second Military Medical University, Shanghai 200438, China; tanyexiong@smmu.edu.cn (Y.T.); 08300700057@fudan.edu.cn (T.J.); dlw@smmu.edu.cn (L.D.)

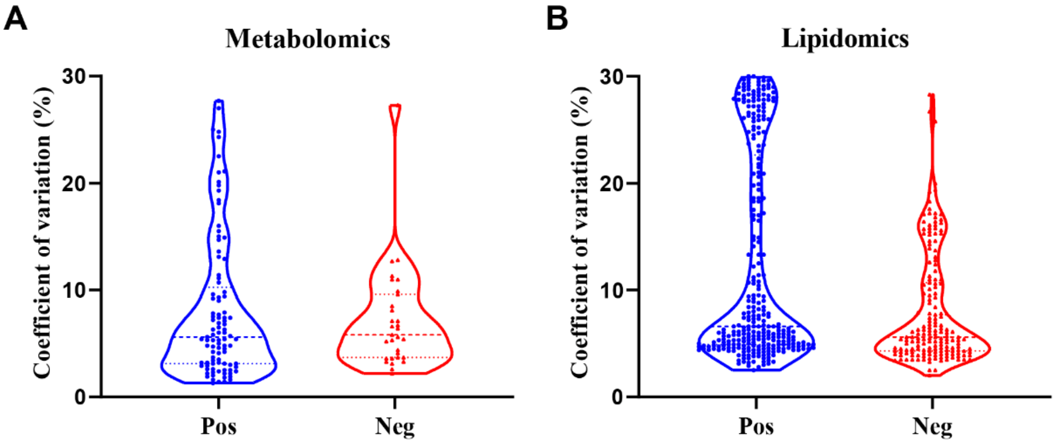

Figure S1. Violin plots of the CV distribution in QC samples from metabolomics (A) and lipidomics (B) sets, respectively.

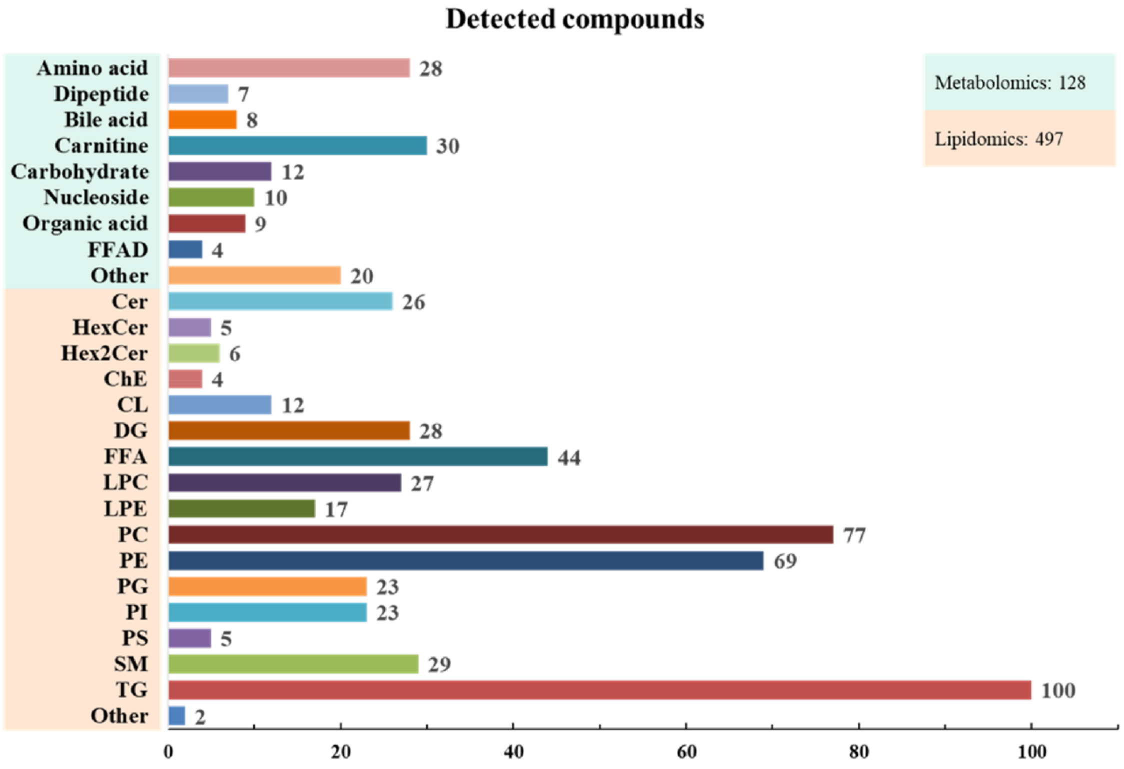

Figure S2. Number of compounds detected in metabolomics and lipidomics data from tissue samples of HCC patients.

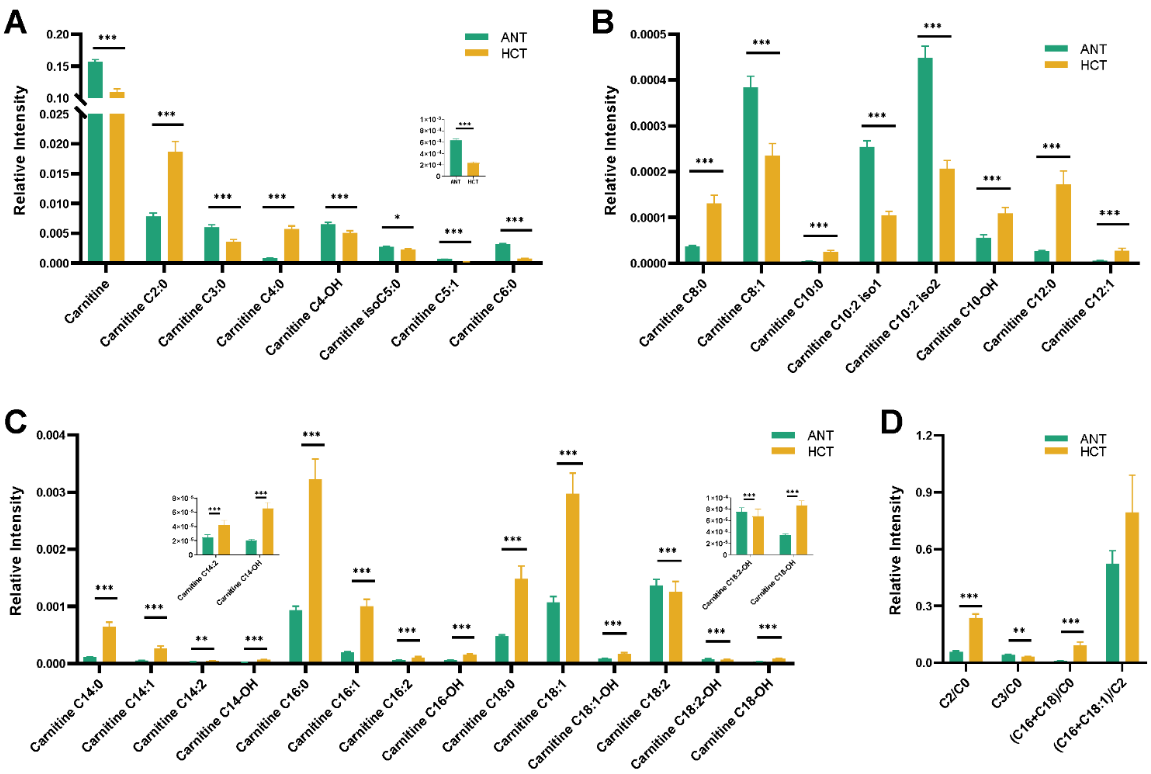

Figure S3. Acylcarnitine metabolism in HCT compared to ANT samples. Bar plots showing the changes of (A) short-chain acylcarnitine, (B) medium-chain acylcarnitine, (C) long-chain acylcarnitine, (D) Carnitine ratios, respectively. * $p<0.05$, ** $p<0.01$, *** $p<0.001$, compared with adjacent noncancerous liver tissue.

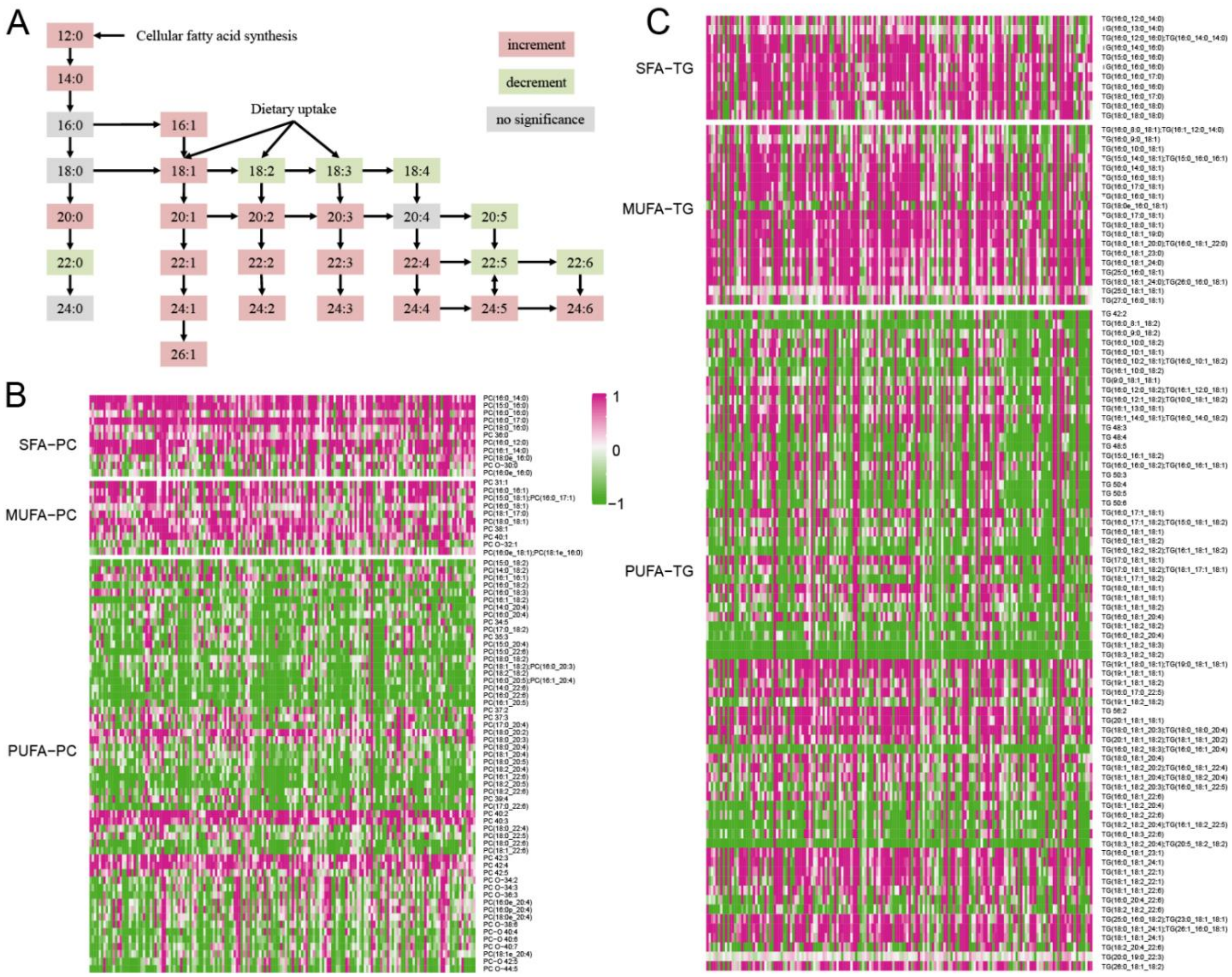

Figure S4. Changes of FFA (A), PC (B) and TG (C) in HCT compared with those in ANT. The fold changes of HCT/ANT were calculated and the log transformed fold-change values were visualized in heatmap (B) and (C).

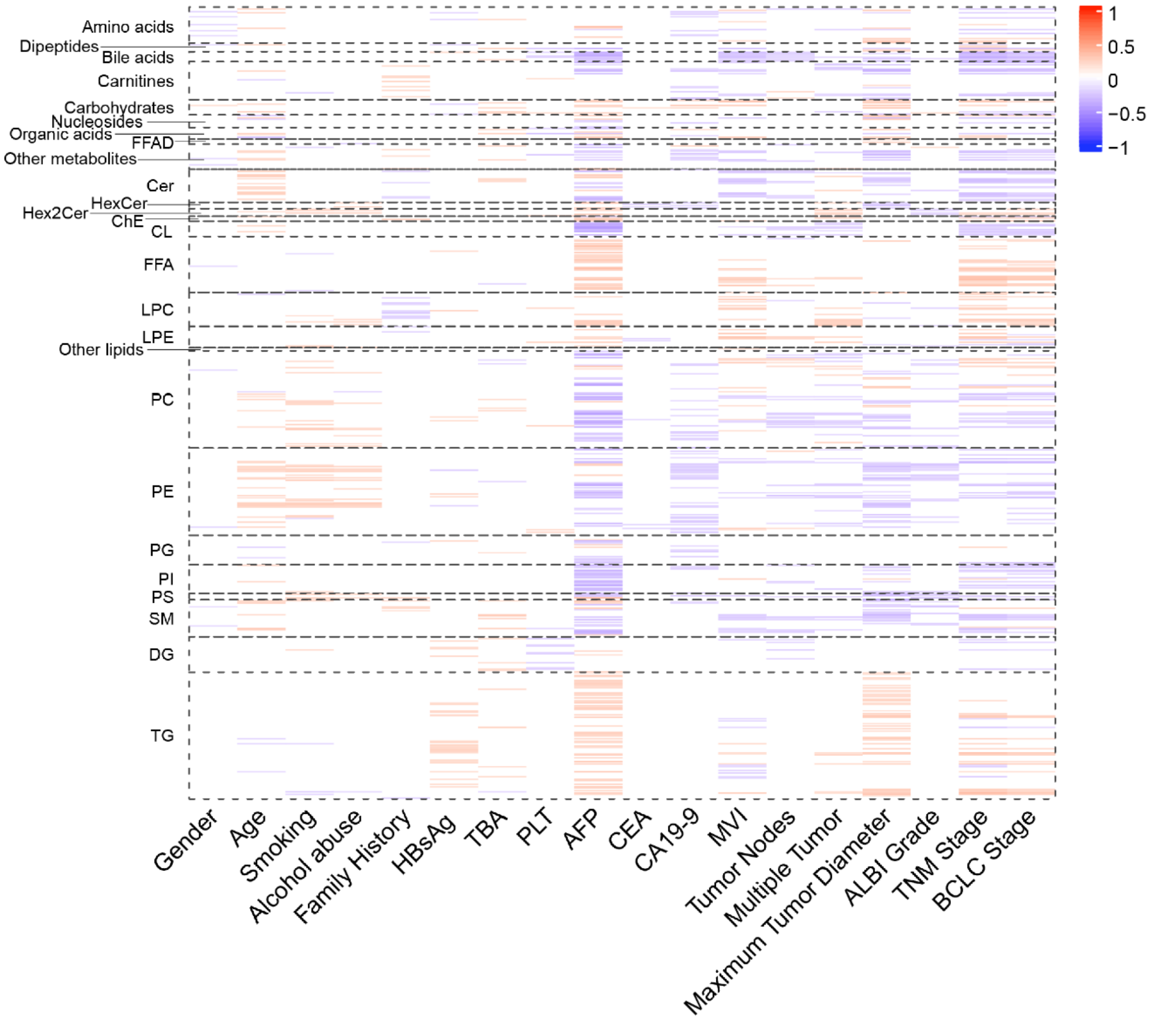

Figure S5. Spearman correlation between the metabolites and clinical factors. The raw p values were adjusted to false discovery rate (FDR) using Benjamini–Hochberg method. The un-significant correlations (p > 0.05) were filled with white.
